# Hand Eczema, Risk Factors and Microbial Skin Contamination in the Norwegian Waste Sorting Industry: A Cross‐Sectional Study

**DOI:** 10.1111/cod.70037

**Published:** 2025-10-26

**Authors:** Jose Hernán Alfonso, Pål Graff, Carla Viegas, Astrid Haaskjold Lossius, Elke Eriksen

**Affiliations:** ^1^ Department of Occupational Medicine and Epidemiology National Institute of Occupational Health, (STAMI) Oslo Norway; ^2^ Department of Dermatology and Venereology Oslo University Hospital, Rikshospitalet Oslo Norway; ^3^ Department of Chemical and Biological Work Environment National Institute of Occupational Health, (STAMI) Oslo Norway; ^4^ Environmental Chemistry Section Norwegian University of Life Sciences Ås Norway; ^5^ H&TRC – Health & Technology Research Center, ESTeSL – Escola Superior de Tecnologia e Saúde Instituto Politécnico de Lisboa Lisbon Portugal; ^6^ NOVA National School of Public Health Public Health Research Centre, Comprehensive Health Research Center, CHRC, NOVA University Lisbon Lisbon Portugal

**Keywords:** bioaerosols, dermatitis, fungi, microorganisms, occupational exposure, personal protective equipment (PPE), skin health, transepidermal water loss (TEWL), waste handling, workplace safety

## Abstract

**Introduction:**

Hand eczema, skin barrier function and skin microbial contamination among waste workers are underexplored. This study aims to assess: (1) the prevalence and risk factors of hand eczema, (2) the levels of transepidermal water loss (TEWL), and (3) skin microbial contamination in waste sorting workers.

**Methods:**

Using the Nordic Occupational Skin Questionnaire—2002, data were collected from 69 waste sorting workers and 25 office personnel. TEWL was measured with a Tewameter (TM 300, Courage+ Khazaka Electronic, Köln). Microbial skin samples were collected from the left dorsal hand with sterile swabs (Copan, Italy) and cultured. Analyses included descriptive statistics and multivariate logistic regression.

**Results:**

The hand eczema prevalence was 25% among waste workers and 40% in office personnel. The prevalence of hand eczema was significantly lower among exposed workers compared to controls (*p* = 0.012). TEWL and fungal concentrations were comparable across groups. Atopic dermatitis (AD) and nicotine use were significant predictors of HE. The prevalence of hand eczema experienced during the past week was significantly associated with elevated bacterial concentrations (*p* value = 0.05) in both groups.

**Conclusions:**

Waste sorting workers had up to 2.4 times higher prevalence of hand eczema compared with the general population, but a lower prevalence than office workers in the same industry. These findings may reflect a potential healthy worker effect. The potential role of bacterial concentrations in the occurrence of hand eczema warrants further investigation.

## Graphical abstract

Hand eczema (HE) prevalence was 25% in waste workers and 40% in office personnel. Atopic dermatitis and nicotine/snus use predicted hand eczema. Increased bacterial contamination was associated with recent HE.

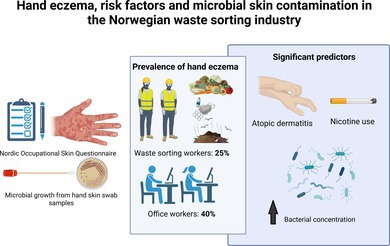

## 1 Introduction

Waste management industries, especially the workforce engaged in waste sorting, are expected to expand throughout the world in order to meet the Sustainable Development Goals (SDGs) established by the United Nations Environment Programme [1]. For example, the European Union's member states currently produce 2.5 billion tons of waste each year, a figure projected to rise to 3.4 billion tons by 2050 [2, 3].

Waste sorting workers, hereafter waste workers, are regularly exposed to biological agents with potential toxic, allergenic and infectious properties during the handling and sorting of waste [4]. Skin problems such as skin rash, infections and even systemic lupus erythematosus have previously been reported in a few studies among waste workers [5, 6]. These studies have shown non‐consistent findings regarding the association between exposure to waste and skin conditions. Recently, technical advancements—such as the implementation of automated waste sorting lines and modifications of waste sorting processes—have introduced new exposure scenarios, highlighting the need to re‐evaluate work risk assessments within the waste industry [7].

Hand eczema (HE) is the most common work‐related skin disease, often resulting from exposure to irritants and allergens in the workplace [8]. Surprisingly, the occurrence of HE among waste workers is still underexplored, despite frequent occupational skin contact with chemical and biological agents [4] potentially compromising skin barrier function. Measurements of skin barrier function in terms of transepidermal water loss (TEWL) have been explored in occupations with a high risk of HE such as health personnel [9], hairdressers [10], cleaners [11], and oil drilling waste workers [12] in order to early identify workers with skin barrier dysfunction and increased risk of developing HE. To what extent this quick and non‐invasive measurement can be used among waste workers at risk of developing HE has not yet been explored.

Microbial skin contamination may play a significant role in skin barrier function and could influence TEWL and the occurrence of HE. The skin microbiome contributes to the maintenance of barrier function and modulates inflammatory responses, potentially influencing the development and severity of skin conditions [13]. Still, the relationship between skin microbiota, barrier function, and the prevalence of HE remains largely unexplored. In a group of waste workers, this study aims to assess (1) the prevalence of HE and its predictors; (2) the usefulness of TEWL measurements for the early identification of workers with skin barrier dysfunction and HE; and (3) the characterisation of skin microbial contamination levels in relation to TEWL levels and the risk of HE among waste workers compared to office personnel.

## 2 Materials and Methods

### 2.1 Sampling Sites and Study Population

**FIGURE 1:**
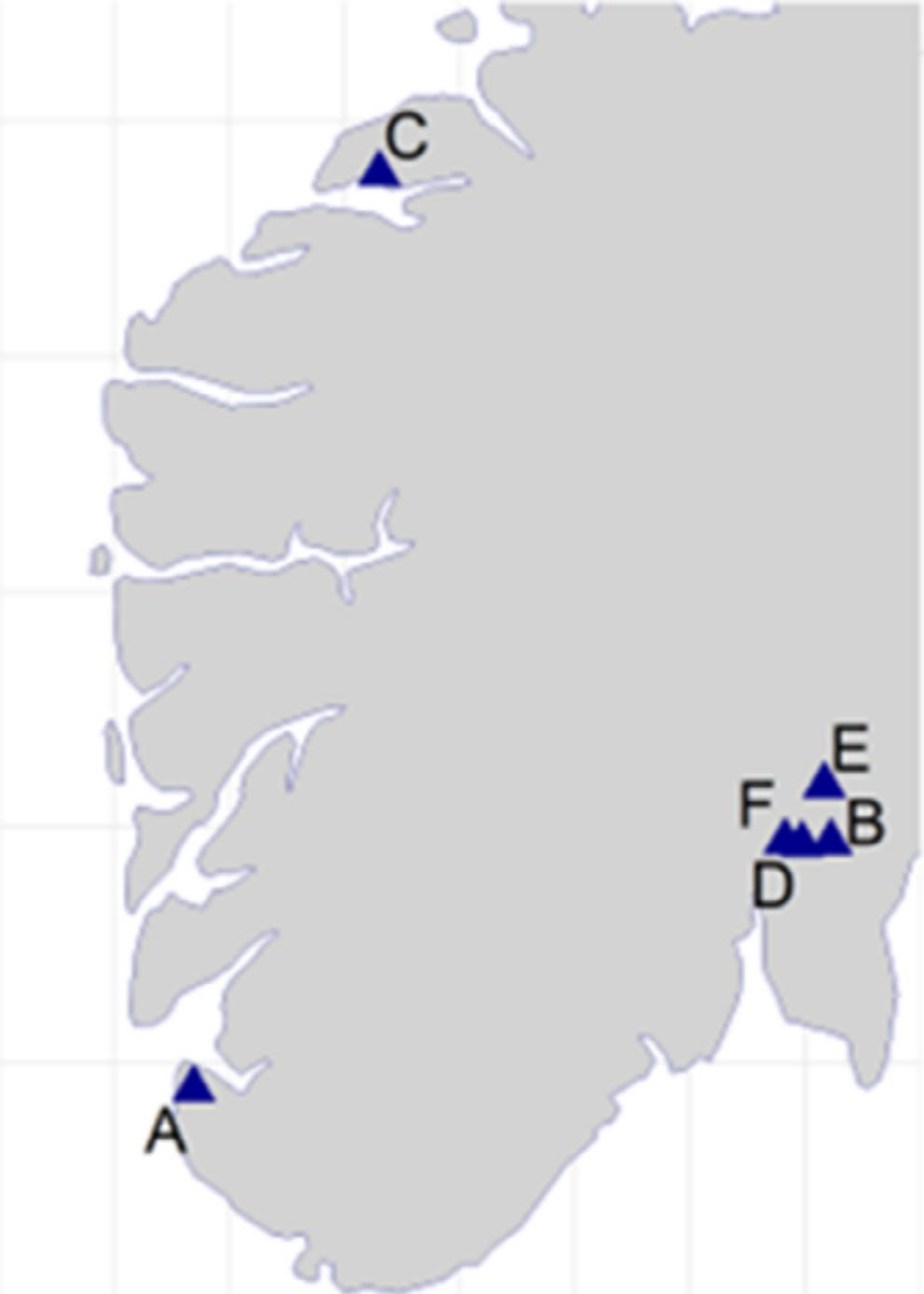
Locations of participating waste sorting plants A–F.

Sampling was conducted at 6 contemporary waste sorting plants (wsp) between June 2020 and November 2021 (Figure 1). Exposed waste workers (*n* = 69) and office personnel (controls, *n* = 25) from the respective plants were invited to participate in the study. Participation was voluntary, and informed consent was obtained prior to participation. At wsp A–C, residual waste was sorted by automated waste sorting lines, whereas at wsp D‐F residual waste from private housing collectives, pre‐sorted plastics and paper/cardboard waste was mainly sorted by hand and excavators.

### 2.2 Questionnaire

An adapted Norwegian version of the Nordic Occupational Skin Questionnaire‐2002 (NOSQ‐2002) [14] was used to collect self‐reported information on workers' skin conditions, including atopic dermatitis, nicotine use, and work‐related data.

### 2.3 TEWL

Skin barrier function was measured in terms of TEWL using a Tewameter (TM 300, Courage + Khazaka Electronic, Köln). Due to the ongoing Covid pandemic and limited access to workers, the TEWL measurement could only be performed on a subset of participants. TEWL measurements were performed on a total of 11 exposed workers and 3 controls working at wsp A (7 exposed, 1 controls) and F (4 exposed, 2 controls).

### 2.4 Microorganisms in Skin Swabs

Skin swabs were collected from exposed waste workers and controls midweek after exposure by swabbing approximately 5cm^2^ of the non‐dominant dorsal hand using sterile Copan eSwabs in liquid Amies medium (ESwab 480C, Copan Diagnostics Inc. CA, USA). The samples were stored at −80 dC until analysis. Skin swabs were cultured on selective media and analysed as described by Viegas et al. [15]. Microbial concentrations were reported as CFU/m^2^. Fungal colonies were identified based on morphology.

### 2.5 Data Analysis

All data analyses were conducted in R/R Studio (version 4.4.1). Statistical analysis was conducted using rstatix [16] and lme4 [17]. Graphs were produced using ggplot2 [18]. The map was created using the sf [19] and rnaturalearth‐package [20]. A logistic regression model was used to study differences in symptom prevalence between exposure groups.

(1) $$\begin{array}{cc} \text{Logit}\left(\text{Prob}\left(\text{eczema}=\mathrm{yes}\right)\right)= & \beta \;\text{atopic eczema}+\text{exposure group}\\ & +\text{smoking habits} \end{array}$$

where atopic eczema (categorical, yes vs. no) and exposure group (categorical, exposed vs. control).

Furthermore, a logistic regression model was used to study the effect of microbial concentrations on the prevalence of skin symptoms, as well as a linear regression model to study the effect of microbial concentration on skin barrier function (TEWL).

(2) $$\text{Logit}\left(\text{Prob}\left(\text{symptom}=\mathrm{yes}\right)\right)=\beta \;\text{bacterial}\;\mathrm{CFU}+\text{fungal}\;\mathrm{CFU}$$

(3) $$\text{Skin barrier function}=\beta_{0}+\beta_{1}\text{bacterial}\;\mathrm{CFU}+\beta_{2}\;\text{fungal}\;\mathrm{CFU}+є$$

Correlation between time spent on clean/unclean work tasks and microbial CFU was reported as Pearson correlation coefficient and Benjamini Hochberg‐corrected *p* values. *p* values below 0.05 were considered statistically significant.

**TABLE 1:** Demographics study population. Split by exposure group and sex range levels in parentheses.

	Controls		Exposed	
	Female	Male	Female	Male
*n* ()	8	17	6	63
Mean age (min–max)	36 (29–41)	45 (31–55)	35 (21–65)	39 (20–65)
Mean BMI (min–max)	25 (22–31)	27 (20–31)	24 (20–32)	27 (20–41)

## 3 Results

### 3.1 Study Population and Questionnaire

#### 3.1.1 Questionnaire

Table 1 shows the demographics of the study population by exposure group and sex (Table 1). The one‐year prevalence of HE was 25% in the exposed population and 40% in the control group (Table 2). In the exposed population, 7% reported HE in association with work, whereas 9% were unsure if it was work‐related. No work‐related HE was reported in the control group. However, 50% of the controls as well as 71% of the exposed workers reported a form of symptom relief during off‐work periods. Atopic eczema was present in 20% of controls and 14% of exposed workers.

**TABLE 2:** Prevalence of risk factors and skin conditions. Figures are given as absolute and relative numbers.

	Controls		Exposed	
	*n*	%	*n*	%
Nicotine (smoking, snus and e‐cigarettes)	5	20	16	23
Atopic eczema	5	20	10	14
Dry hands (past week)	5	20	31	45
Hand eczema (one‐year prevalence)	10	40	17	25
Hand eczema (past week)	1	4	10	14

When comparing workers at plant ABC (automation) and plant DEF (manual sorting), no statistically significant differences in the prevalence of hand eczema were observed in the adjusted model.

Four workers stated that the HE worsened with the use of nitrile or latex gloves. Two workers stated that the HE worsened when in contact with dirt/dust and paper, and one when exposed to cold temperatures.

### 3.2 Models

Significant predictors of HE included atopic eczema (*p* value = 0.04), hand eczema (*p* value = 0.01), and dry hands (*p* value = 0.020) (Table 3). The use of nicotine products was a significant indicator for HE (*p* value = 0.02) and dry hands (*p* value = 0.05). Work‐related eczema as well as dry hands were less prevalent in the exposed group; the differences were, however, not statistically significant (*p* values = 0.08 and 0.06, respectively).

**TABLE 3:** Prevalence of hand eczema in relation to exposure group, atopic eczema and smoking habits.

Predictors	Hand eczema (1 year)		Work‐related eczema		Hand eczema (past week)		Dry hands (past week)	
	OR	*p*	OR	*p*	OR	*p*	OR	*p*
Intercept	0.77	0.5	0.08	0.00	0.12	0.00	0.14	0.00
Status (ref.: control)	0.54	0.25	3.99	*0.08*	1.59	0.54	3.04	*0.06*
Atopic eczema (ref.: no)	4.07	**0.04**	2.17	0.27	5.90	**0.01**	5.24	**0.02**
Nicotine use (ref.: no)	0.27	**0.02**	0.75	0.61	0.24	0.08	2.51	**0.05**
Observations	94		94		94		94	

*Note*: Statistically significant *p* values (= /< 0.05) in bold.

### 3.3 Microbial Concentrations in Skin Swabs

Average bacterial CFU concentrations and skin barrier measurements were comparable between the exposure groups (Table 4). There were relatively low fungal CFU concentrations in the samples, whereas bacterial counts were higher. Gram‐negative bacteria were identified in samples collected from exposed workers; however, they were absent in samples from controls. Among fungi, Penicillium sp. was generally the most prevalent genus identified in skin swab samples, except from samples collected in controls at wsp A, in which Cladosporium sp. was dominant. In samples collected from exposed workers at wsp B, *A. nigri* and *A. fumigatus* were identified. Detailed results can be found elsewhere [15].

**TABLE 4:** Average CFU counts (AM) and skin barrier measurements by exposure group.

	*n*	Bacterial CFU/m^2^	Fungal CFU/m^2^	TEWL
Control	27	7.4 × 10^6^	5.0 × 10^3^	22.2
Exposed	73	8.7 × 10^6^	7.7 × 10^3^	18.5

The prevalence of HE experienced during the past week was significantly associated with elevated bacterial concentrations (*p* value = 0.05) (Table 5). Bacterial CFU concentrations were significantly negatively associated with reduced skin barrier levels (*p* value = 0.019), the effect was, however very low. Bacterial concentrations did not affect the prevalence of dry hands during past week. Fungal concentrations were not a contributing factor to any of the assessed skin conditions.

**TABLE 5:** Model output skin barrier function (TEWL), 1‐year prevalence of hand eczema, hand eczema during the past week, and dry hands during the past week as response variables in association with bacterial and fungal CFU concentrations in personal skin‐biota swabs across exposure groups.

Predictors	Hand eczema		Hand eczema (past week)		Dry hands (past week)		TEWL	
	OR	*p*	OR	*p*	OR	*p*	OR	*p*
Intercept	0.47	0.17	0.05	0.00	0.76	0.61	45.32	0.00
Fungal CFU	1.00	0.37	1.00	0.42	1.00	0.86	0.00	0.22
Bacterial CFU	1.00	0.27	1.00	**0.05**	1.00	0.91	−0.00	**0.01**
Observations	30		29		29		8	

*Note*: Statistically significant *p* values (= /< 0.05) in bold.

**FIGURE 2:**
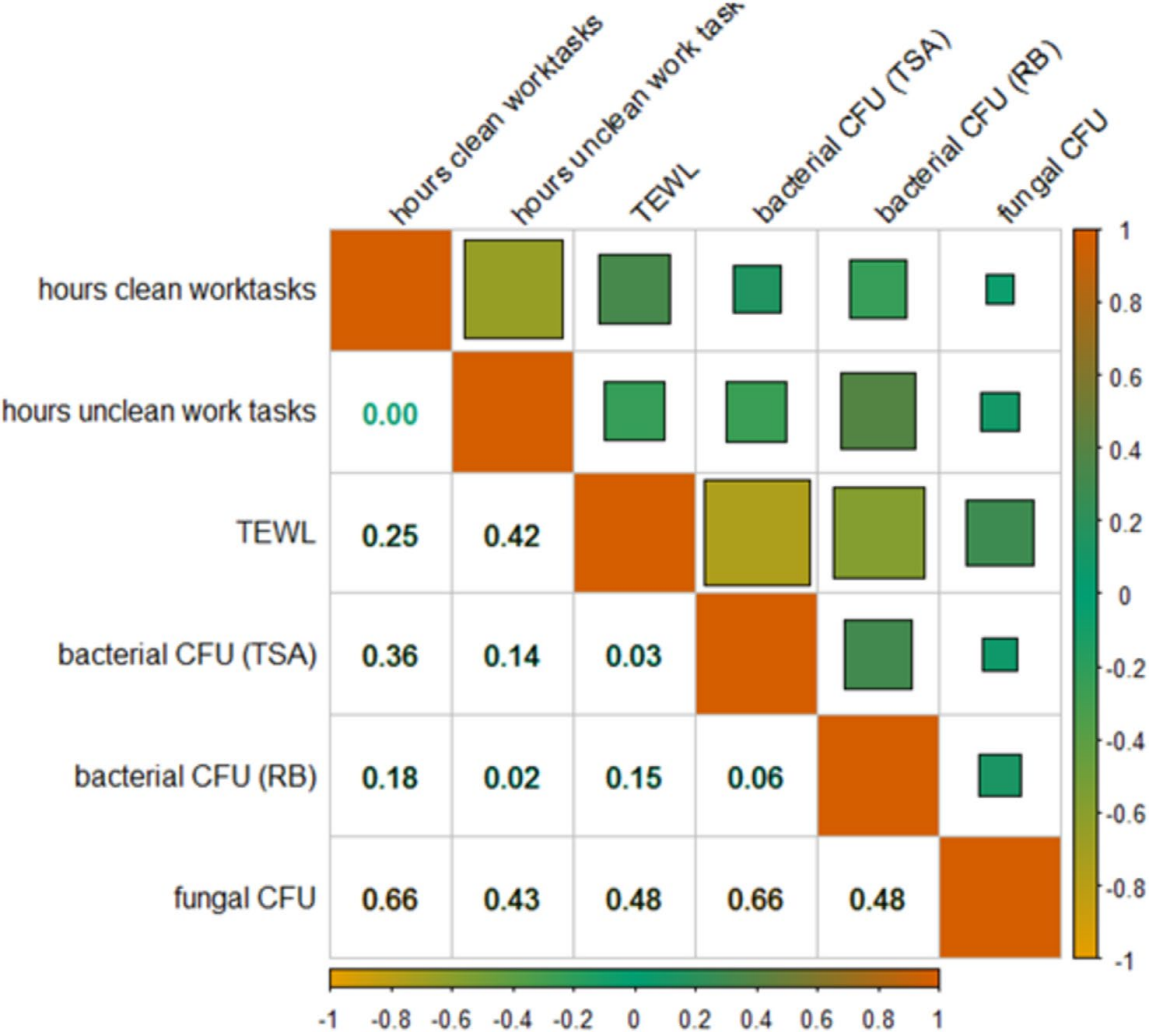
Correlation between time spent on clean and unclean work tasks and bacterial and fungal CFUs in skin swab samples. Upper right corner colour coded Pearson correlation coefficients, lower left corner Benjamini Hochberg corrected *p* values. The strength of the correlation is indicated in the colour gradient.

TEWL levels were significantly negatively associated with increased bacterial concentrations (*p* value = 0.03) (Figure 2). Time spent on unclean work tasks was significantly negatively associated with bacterial CFUs (*p* value = 0.02).

## 4 Discussion

The prevalence of HE among waste workers was 2.4 times higher, and work‐related HE was three times higher than in the general Norwegian population [21]. Interestingly, it was unexpectedly lower than in office workers who exhibited a 1.5‐fold higher HE prevalence than waste workers, and up to 3.6 times higher than the general population.

A potential “healthy worker survivor” effect may have influenced our findings, as previous evidence at the populational level suggests that skin problems are associated with change of jobs in Norway's general workforce [22]. Specifically, some individuals with pre‐existing skin issues may have transitioned from waste sorting to office roles, resulting in a selection bias—an example of the healthy worker effect under employment. Conversely, workers with prior skin problems could also have been allocated to less exposed roles from the outset [22].

It is noteworthy that, during data collection amid the COVID‐19 pandemic, 50% of controls and 71% of exposed workers reported relief from skin problems during off‐work periods. We hypothesize that heightened hygiene measures—such as required frequent handwashing and sanitizer use at work—may have contributed to these observations [23]. Most workers at various plants reported high usage of both soap and hand sanitizer throughout their shifts. For instance, the detection of Gram‐negative bacteria exclusively among exposed workers, together with the identification of *A. fumigatus* and *A. nigri* in samples from WSP B, suggests an occupationally related influence on the skin microbiota. In contrast, the predominance of Cladosporium in control samples likely reflects background environmental exposure, while the frequent presence of Penicillium across groups may represent ubiquitous environmental contamination rather than a specific occupational signal [15]. A further exploratory study on dysbiosis and diversity of the skin microbiota in this occupational setting is in preparation [24].

Our study reinforces that workers with atopic eczema are at higher risk for HE [24] and that nicotine use is a risk factor for HE [25]. It underscores the importance of focusing on these individuals when it comes to primary and secondary prevention strategies to reduce the occurrence and severity of HE.

Interestingly, glove use did not significantly influence HE prevalence among waste workers, suggesting that reliance solely on personal protective equipment may be insufficient. This highlights the need for technical and organisational interventions that effectively reduce hazardous exposures, as these measures most likely have more impact than protective measures alone [26].

We observed indications of a possible association between skin microbial contamination and HE occurrence. Our data showed a modest yet significant association between elevated bacterial levels and recent HE, suggesting a potential role for bacteria in skin health among workers. Skin colonisation with *Staphylococcus aureus* has been linked to both atopic hand eczema and increased severity for HE [27], and individuals with HE may develop a more dysbiotic skin bacterial community over time [28]. Further research focused on microbial diversity and community structure is ongoing to better understand the involvement of bacteria in HE within this occupational group [29]. Notably, we observed a significant negative association between the time spent on unclean work tasks and bacterial levels, which might imply that certain contaminated tasks are associated with decreased bacterial colonisation—possibly due to cleaning protocols, workload patterns, use of protective equipment or other factors.

The lack of significant differences in TEWL measurements between waste and office workers is likely related to the limited number of observations. Future studies with larger sample sizes are needed to clarify the potential usefulness of TEWL measurements for assessing skin barrier function in this occupational setting.

### 4.1 Limitations

Due to the cross‐sectional design of this study, the findings are exploratory and do not establish causal relationships. Reliance on self‐reported data may introduce bias; however, existing evidence suggests that self‐reports of HE tend to underestimate rather than overestimate the true prevalence [30, 31, 32]. Thus, any potential bias is more likely to have led to the underestimation of the actual occurrence. Furthermore, self‐reporting limited our ability to determine the aetiology of HE cases or to classify them morphologically.

The survey and sampling occurred during the COVID‐19 pandemic, which prompted widespread hygiene measures—including increased handwashing and sanitizer use—potentially affecting skin health. Nevertheless, we do not expect these pandemic‐related behavioural changes to have differentially influenced waste workers compared to office workers.

### 4.2 Implications and Relevance

The practical implications of this study include:

‐Assessment of biological exposures: biological exposures should be routinely evaluated in occupational dermatology research and incorporated into primary prevention strategies, alongside physical and chemical hazards. Our previous research demonstrated significant associations between self‐reported skin exposure to biological agents and long‐term sick leave in the Norwegian working population [33]. The skin, as the body's largest organ, plays a dynamic role—both interacting with and defending against environmental and occupational mechanical, physical, chemical, and biological exposures. Currently, the Norwegian workforce employed in waste handling is around 11 024 workers, and the latest statistics showed that the number of workers who are regularly exposed to biological material is steadily growing [34]. Future studies should consider including an additional control group when a potential healthy worker effect within the same occupational setting is suspected.

‐Inclusive prevention strategies: Occupational groups with minimal chemical or physical exposures—such as office workers—should not be overlooked in prevention strategies. In our study, these individuals also reported HE, possibly related to pre‐existing skin conditions or workplace accommodations following earlier skin problems in either waste sorting plant employment or potentially exposure in other occupations with increased risk for HE.

### 4.3 Conclusions

This study highlights that workers in the waste sorting industry should be considered a risk group for HE. While this sector poses substantial risks to skin health, the observed prevalence may be underestimated because of a potential healthy worker effect. Atopic eczema and nicotine use were predictors of HE, and bacterial levels may play a role in the development of HE. Targeted interventions are needed to reduce the burden of HE among waste and office workers in the Norwegian waste sorting industry.

## Author Contributions

**J.H.A.:** conceptualization, methodology, data analysis, writing – original draft, writing – review and editing. **E.E.:** conceptualization, investigation, data analysis, writing – original draft, writing – review and editing. **C.V.:** writing – review and editing, data analysis, writing – review and editing. **A.H.L.:** methodology, writing – review and editing. **P.G.:** conceptualization, writing – review and editing, supervision, funding acquisition, project administration.

## Ethics Statement

This study has been approved by the Regional Ethics Committee in Oslo, REC South‐East B (ref. no.: 34312).

## Consent

Informed consent was obtained from all individual participants.

## Conflicts of Interest

Jose Hernán Alfonso and Astrid Haaskjold Lossius have received an unrestricted research grant and honoraria for presentations from Sanofi. Jose Hernán Alfonso has received honoraria for presentations from Almirall. Astrid Haaskjold Lossius has received honoraria for presentations from Sanofi, Abbvie and Novartis. She participated in Advisory Boards for Abbvie and UCB. The other authors declare no conflicts of interest.

## Data Availability

The data that support the findings of this study are available on request from the corresponding author. The data are not publicly available due to privacy or ethical restrictions.

## References

[1] United Nations Environment Programme , Global Waste Management Outlook 2024: Beyond an Age of Waste–Turning Rubbish Into a Resource (UNEP, 2024), https://wedocs.unep.org/20.500.11822/44939.

[2] European Commission , A New Circular Economy Action Plan for a Cleaner and More Competitive Europe (European Commission, 2020), https://eur‐lex.europa.eu/legal‐content/EN/TXT/?uri=celex:52020DC0098.

[3] M. Negrete‐Cardoso , G. Rosano‐Ortega , E. L. Álvarez‐Aros , M. E. Tavera‐Cortés , C. A. Vega‐Lebrún , and F. J. Sánchez‐Ruíz , “Circular Economy Strategy and Waste Management: a Bibliometric Analysis in Its Contribution to Sustainable Development, Toward a Post‐COVID‐19 Era,” Environmental Science and Pollution Research International 29 (2022): 61729–61746, 10.1007/s11356-022-18703-3. 35668274 PMC9170551

[4] E. Eriksen , P. Graff , I. Pedersen , A. Straumfors , and A. K. Afanou , “Bioaerosol Exposure and In Vitro Activation of Toll‐Like Receptors in a Norwegian Waste Sorting Plant,” Safety and Health at Work 13 (2022): 9–16, 10.1016/j.shaw.2021.09.002. 35936194 PMC9349000

[5] O. M. Poulsen , N. O. Breum , N. Ebbehøj , et al., “Collection of Domestic Waste. Review of Occupational Health Problems and Their Possible Causes,” Science of the Total Environment 170, no. 1–2 (1995): 1–19, 10.1016/0048-9697(95)04524-5. 7569875

[6] M. Megna , M. Napolitano , C. Costa , N. Balato , and C. Patruno , “Waste Exposure and Skin Diseases,” Giornale Italiano di Dermatologia e Venereologia 152, no. 4 (2017): 379–382, 10.23736/s0392-0488.17.05505-5. 28209048

[7] E. Eriksen , A. K. Afanou , A. M. Madsen , A. Straumfors , and P. Graff , “An Assessment of Occupational Exposure to Bioaerosols in Automated Versus Manual Waste Sorting Plants,” Environmental Research 218 (2023): 115040. 36521541 10.1016/j.envres.2022.115040

[8] J. P. Thyssen , M. L. A. Schuttelaar , J. H. Alfonso , et al., “Guidelines for Diagnosis, Prevention, and Treatment of Hand Eczema,” Contact Dermatitis 86, no. 5 (2022): 357–378, 10.1111/cod.14035. 34971008

[9] Ž. Babić , F. Šakić , I. J. Rapić , L. Lugović‐Mihić , and J. Macan , “Difference Between Hand and Forearm Transepidermal Water Loss and Skin pH as an Improved Method to Biomonitor Occupational Hand Eczema: Our Findings in Healthcare Workers,” Arhiv za Higijenu Rada i Toksikologiju 75, no. 3 (2024): 172–179, 10.2478/aiht-2024-75-3885. 39369331 PMC11456224

[10] Ž. Babić , T. Samardžić , and J. Macan , “Comparison of Beautician and Hairdressing Apprentices With Regard to Skin Health and Skin Barrier Function,” Arhiv za Higijenu Rada i Toksikologiju 71, no. 3 (2020): 190–196, 10.2478/aiht-2020-71-3452. 33074168 PMC7968498

[11] J. Douwes , T. Slater , M. Shanthakumar , et al., “Determinants of Hand Dermatitis, Urticaria and Loss of Skin Barrier Function in Professional Cleaners in New Zealand,” International Journal of Occupational and Environmental Health 23, no. 2 (2017): 110–119, 10.1080/10773525.2018.1427307. 29359638 PMC6060852

[12] J. H. Alfonso , R. Olsen , P. Graff , et al., “O5B.1 Workplace Exposure Assessment (WEA), skin Barrier Function, and Occurrence of Hand Eczema Among Workers Handling Drilling Waste in Norway,” Occupational and Environmental Medicine 76 (2019): A43‐A43, 10.1136/OEM-2019-EPI.116.

[13] X. E. Zhang , P. Zheng , S. Z. Ye , et al., “Microbiome: Role in Inflammatory Skin Diseases,” Journal of Inflammation Research 17 (2024): 1057–1082, 10.2147/JIR.S441100. 38375021 PMC10876011

[14] P. Susitaival , M. A. Flyvholm , B. Meding , et al., “Nordic Occupational Skin Questionnaire (NOSQ‐2002): a New Tool for Surveying Occupational Skin Diseases and Exposure,” Contact Dermatitis 49, no. 2 (2003): 70–76, 10.1111/j.0105-1873.2003.00159.x. 14641353

[15] C. Viegas , E. Eriksen , B. Gomes , et al., “Comprehensive Assessment of Occupational Exposure to Microbial Contamination in Waste Sorting Facilities From Norway,” Frontiers in Public Health 11 (2023): 1297725. 38179569 10.3389/fpubh.2023.1297725 PMC10766354

[16] A. Kassambara , “rstatix: Pipe‐Friendly Framework for Basic Statistical Tests,” 2022, https://CRAN.R‐project.org/package=rstatix.

[17] D. Bates , M. Maechler , B. Bolker , and S. Walker , “Fitting Linear Mixed‐Effects Models Using lme4,” Journal of Statistical Software 67, no. 1 (2015): 1–48, 10.18637/jss.v067.i01.

[18] H. Wickham , ggplot2: Elegant Graphics for Data Analysis (Springer‐Verlag, 2016), https://ggplot2.tidyverse.org.

[19] E. Pebesma and R. Bivand , Spatial Data Science: With Applications in R (Chapman and Hall/CRC, 2023), 10.1201/9780429459016.

[20] P. Massicotte and A. South , “rnaturalearth: World Map Data From Natural Earth,” 2023, https://CRAN.R‐project.org/package=rnaturalearth.

[21] H. K. Vindenes , C. Svanes , S. H. L. Lygre , B. E. Hollund , A. Langhammer , and R. J. Bertelsen , “Prevalence of, and Work‐Related Risk Factors for, Hand Eczema in a Norwegian General Population (The HUNT Study),” Contact Dermatitis 77, no. 4 (Octember 2017): 214–223, 10.1111/cod.12800. 28449354

[22] J. H. Alfonso and H. A. Johannessen , “Self‐Reported Skin Problems and the Healthy Worker Effect in the General Working Population of Norway: a Three‐Year Prospective Study,” Scandinavian Journal of Work, Environment & Health 45, no. 5 (September 2019): 450–457, 10.5271/sjweh.3810. 30826843

[23] H. K. Vindenes , R. J. Bertelsen , S. H. L. Lygre , T. Morken , O. J. Møllerløkken , and K. Irgens‐Hansen , “Changes in Infection Prevention Practices and Occurrence of Skin Symptoms Among Healthcare Workers, Cleaners and Day‐Care Workers in Norway During the COVID‐19 Pandemic,” Acta Dermato‐Venereologica 103 (2023): adv00840, 10.2340/actadv.v103.3420. 36600529 PMC9885284

[24] S. M. D. Ruff , K. A. Engebretsen , C. Zachariae , et al., “The Association Between Atopic Dermatitis and Hand Eczema: a Systematic Review and Meta‐Analysis,” British Journal of Dermatology 178, no. 4 (April 2018): 879–888, 10.1111/bjd.16147. 29172235

[25] K. A. Zimmer , E. S. Armbrecht , and N. M. Burkemper , “The Association of Smoking With Contact Dermatitis and Hand Eczema–A Review,” International Journal of Dermatology 57, no. 4 (April 2018): 375–387, 10.1111/ijd.13777. 28960277

[26] J. H. Alfonso , A. Bauer , L. Bensefa‐Colas , et al., “Minimum Standards on Prevention, Diagnosis and Treatment of Occupational and Work‐Related Skin Diseases in Europe: Position Paper of the COST Action StanDerm (TD 1206),” Journal of the European Academy of Dermatology and Venereology 31, no. Suppl 4 (2017): 31–43, 10.1111/jdv.14319. 28656728

[27] L. B. Nørreslet , S. M. Edslev , P. S. Andersen , et al., “Colonization With *Staphylococcus aureus* in Patients With Hand Eczema: Prevalence and Association With Severity, Atopic Dermatitis, Subtype and Nasal Colonization,” Contact Dermatitis 83, no. 6 (December 2020): 442–449, 10.1111/cod.13679. 32720317

[28] H. K. Vindenes , C. Drengenes , H. Amin , K. Irgens‐Hansen , C. Svanes , and R. J. Bertelsen , “Longitudinal Analysis of the Skin Microbiome in Association With Hand Eczema, Hand Hygiene Practices and Moisturizer Use,” Journal of the European Academy of Dermatology and Venereology 38, no. 11 (November 2024): 2118–2129, 10.1111/jdv.19906. 38419413

[29] E. Eriksen , P. Graff , A. H. Lossius , and J. H. Alfonso , “Skin Microbiota Dysbiosis and Diversity in Waste Sorting Workers,” (In preparation).

[30] H. A. Smit , P. J. Coenraads , A. P. Lavrijsen , and J. P. Nater , “Evaluation of a Self‐Administered Questionnaire on Hand Dermatitis,” Contact Dermatitis 26, no. 1 (1992): 11–16, 10.1111/j.1600-0536.1992.tb00861.x. 1600733

[31] P. Susitaival , L. Husman , A. Hollmén , and M. Horsmanheimo , “Dermatoses Determined in a Population of Farmers in a Questionnaire‐Based Clinical Study Including Methodology Validation,” Scandinavian Journal of Work, Environment & Health 21, no. 1 (1995): 30–35, 10.5271/sjweh.5. 7784862

[32] P. Susitaival , “Questionnaire Methods in Occupational Skin Disease Epidemiology,” in Kanerva's Occupational Dermatology, ed. S. John , J. Johansen , T. Rustemeyer , P. Elsner , and H. Maibach (Springer, 2020), 10.1007/978-3-319-68617-2_81.

[33] J. H. Alfonso , T. Tynes , J. P. Thyssen , J. Ø. Holm , and H. A. Johannessen , “Self‐Reported Occupational Skin Exposure and Risk of Physician‐Certified Long‐Term Sick Leave: A Prospective Study of the General Working Population of Norway,” Acta Dermato‐Venereologica 96, no. 3 (2016): 336–340, 10.2340/00015555-2253. 26439508

[34] Norwegian Institute of Occupational Health , Faktabok om arbeidsmiljø og helse 2024 (STAMI, 2024), https://noa.stami.no/wp‐content/uploads/2024/09/Faktabok‐om‐arbeidsmiljo‐og‐helse‐2024.pdf.

